# Association between the newly proposed Chinese dietary inflammatory index and risk of ischemic stroke: a multi-centered case—control study

**DOI:** 10.3389/fnut.2026.1861328

**Published:** 2026-07-22

**Authors:** Xiyu Chen, Huacheng Xu, Zhen Feng

**Affiliations:** 1 Department of Neurology, Yichang Central People’s Hospital, Yichang, Hubei, China; 2 Department of Cardiovascular Medicine, Zaoyang First People's Hospital, Zaoyang, Hubei, China; 3 Department of Pre-Hospital Emergency Care, Shenzhen Airport Medical Emergency Center, Shenzhen, Guangdong, China

**Keywords:** case–control studies, dietary inflammatory index, inflammation, ischemic stroke, oxidative stress

## Abstract

**Background:**

Inflammation is a key mechanism in ischemic stroke pathogenesis. This study investigates the association between the Chinese Dietary Inflammatory Index (CHINA-DII) and ischemic stroke risk in a Chinese population.

**Methods:**

In this multi-centered hospital-based case–control study, 292 Chinese patients with first-ever ischemic stroke were compared with 292 age- and sex-matched controls. The CHINA-DII was calculated based on dietary intakes linked to inflammation, including specific nutrients and foods that contribute to pro-inflammatory or anti-inflammatory pathways. Multivariable logistic regression was used to calculate odds ratios (ORs) and 95% confidence intervals (CIs) for CHINA-DII percentiles, adjusting for key potential confounders.

**Results:**

Compared to controls, stroke patients had a significantly higher CHINA-DII scores than controls (−1.16 ± 1.01 vs. −1.88 ± 0.95, *p* < 0.001). In multivariable-adjusted logistic regression analyses, each one-unit increase in CHINA-DII score was associated with a 27% higher odds of stroke (OR: 1.27; 95% CI: 1.10–1.46). Compared with participants in the lowest tertile of CHINA-DII, those in the highest tertile had 2.41-fold greater odds of stroke (OR: 2.41; 95% CI: 1.51–3.84; P for trend = 0.001).

**Conclusion:**

A higher CHINA-DII score is associated with an increased risk of ischemic stroke in this Chinese cohort. While dietary inflammatory factors may contribute to stroke risk, the role of individual dietary antioxidants in stroke prevention remains uncertain.

## 1 Introduction

Stroke is a severe cerebrovascular event and a major global health concern, with approximately 12.2 million new cases and 101 million prevalent cases reported in 2019 (1). In China, ischemic stroke represents the predominant subtype of stroke and contributes substantially to the national burden, with age-standardized prevalence estimates for ischemic stroke exceeding 1,000 cases per 100,000 people in recent population surveys, making it a major public health issue (2). Ischemic stroke is a neurological condition caused by an abrupt reduction or blockage of cerebral blood flow, leading to oxygen deprivation and subsequent neuronal injury (3). Well-established and modifiable risk factors for this neurovascular event encompass smoking, physical inactivity, obesity, hypertension, diabetes, cardiovascular disease, and an unhealthy diet (4). Consequently, implementing effective preventive measures is crucial to reduce the impact of this condition.

Inflammation is an essential biological response to injury or infection, but when dysregulated, it contributes to vascular dysfunction, endothelial damage, and atherosclerosis, all of which are critical in the development of ischemic stroke (5). Reactive oxygen species (ROS) generated during inflammation further exacerbate oxidative stress, promoting endothelial dysfunction and vascular injury (6). Chronic low-grade inflammation has been implicated in the progression of ischemic stroke (7), and this inflammatory response can be influenced by dietary factors, particularly through the intake of pro-inflammatory and anti-inflammatory foods (8).

The Chinese Dietary Inflammatory Index (CHINA-DII) is a newly proposed tool designed to assess the inflammatory potential of an individual’s diet based on food and nutrient consumption patterns (9). The CHINA-DII incorporates a wide range of foods and nutrients that either promote or reduce systemic inflammation, providing a more tailored index for the Chinese population (9). Diets rich in pro-inflammatory foods (e.g., red meat, refined grains, and sugary beverages) tend to increase the CHINA-DII score, while diets high in anti-inflammatory components (e.g., fruits, vegetables, and fish) contribute to a lower score (9). The CHINA-DII has gained attention as a promising marker for studying the relationship between diet-induced inflammation and chronic diseases (10, 11).

Emerging studies have demonstrated the association between diet-induced inflammation and stroke risk, with several showing that higher inflammatory diets (as reflected by higher DII scores) are linked to an increased risk of ischemic stroke (12, 13). This study extends previous research on dietary inflammatory indices by applying the newly developed CHINA-DII, which is specifically tailored to Chinese dietary patterns, to investigate ischemic stroke risk. Unlike prior studies that primarily relied on Western-based indices, our study provides novel evidence from a Chinese population using a hospital-based case–control design focused on first-ever ischemic stroke.

## 2 Methods

### 2.1 Study design and setting

This 1:1 multi-centered hospital-based case–control study was designed to examine the association between the CHINA-DII and the risk of ischemic stroke. The study was conducted at Yichang Central People’s Hospital and Zaoyang First People’s Hospital from March 2020 to April 2023.

Ethical approval was obtained from the Yichang Central People’s Hospital (Ethics code: 2020-122-05) and Zaoyang First People’s Hospital (Ethics code: 2020-078-21), This study was conducted in accordance with the principles of the Declaration of Helsinki, and all participants provided written informed consent prior to enrollment. The study protocol adhered to the guidelines of the Strengthening the Reporting of Observational Studies in Epidemiology (STROBE) statement.

### 2.2 Participants

Participants were recruited from Neurology Department at Yichang Central People’s Hospital and Zaoyang First People’s Hospital, and cases comprised newly diagnosed (≤1 month prior to the interview), clinically and radiologically confirmed (by Craniocerebral CT or MRI) ischemic stroke patients aged 18 years and older. Cases were eligible if they presented within 5 days of symptom onset or within 72 h of hospital admission, and if a CT or MRI of the brain was planned within 1 week of presentation (14). For patients unable to communicate sufficiently to complete the study questionnaire, a proxy respondent (spouse or first-degree relative living in the same household and aware of the patient’s medical history) was used. Controls were selected from the same outpatient departments of hospitals and matched to cases by age (±5 years), gender, and ethnicity, with no history of stroke.

Exclusion criteria included patients with non-vascular causes of stroke, hemorrhagic stroke, those currently hospitalized for acute coronary syndrome, individuals with chronic diseases affecting diet or metabolism (e.g., liver diseases, renal failure), individuals who experienced serious trauma or underwent major surgery within the month prior to the stroke event, pregnant or lactating women, individuals on antioxidant supplementation, or if consent could not be obtained from the patient or a valid proxy respondent (14).

### 2.3 Sample size calculation

The required sample size was calculated using power analysis to detect a significant association between the CHINA-DII and ischemic stroke risk. Based on findings from a previous study examining the relationship between a dietary index and gastric cancer (15), assuming an odds ratio of 0.6, a power of 90%, and a significance level of 0.05, it was estimated that 292 cases and 292 controls per group would be sufficient to detect a meaningful difference.

### 2.4 Baseline interviews and measurements

Demographic and clinical data, including age, gender, body mass index (BMI), smoking status, alcohol consumption, physical activity, income level, and family history of stroke, were collected through structured interviews and medical record reviews. Smoking status was classified as current smokers (individuals who smoke regularly, at least one cigarette per day during the year of interview or the preceding year) and never smokers (individuals who have never smoked). Alcohol consumption was defined as intake of at least one alcoholic beverage per day for a minimum of six consecutive months. Physical activity was characterized as engaging in moderate to vigorous exercise for at least 30 min per session. Weight was measured using a calibrated scale with participants standing upright, barefoot, and wearing minimal clothing. Height was measured with a stadiometer while the participant stood straight with the head in the Frankfurt plane, and BMI was calculated by dividing weight (kg) by height squared (m^2^).

### 2.5 Calculation of China dietary inflammatory index (CHINA-DII) score

Dietary intake data were collected using a validated semi-quantitative 149-item Chinese-formatted, culturally adapted Food Frequency Questionnaire (FFQ) (16), administered by trained interviewers. To improve the accuracy of dietary intake assessment, the FFQ was supplemented with 24-h dietary recalls. The daily consumption of nutrients was estimated utilizing data from the 2009 edition of the Chinese Food Composition Table (17).

The CHINA-DII was calculated based on the newly developed and validated method by Chen et al., which involves scoring dietary components according to their inflammatory potential (9). The CHINA-DII was computed using 27 food component, including energy, protein, carbohydrate, total fat, saturated fatty acids, monounsaturated fatty acids, polyunsaturated fatty acids, cholesterol, dietary fiber, folate, vitamins A, B1, B2, B3, B6, B12, C, D, and E, *β*-carotene, iron, zinc, magnesium, selenium, calcium, caffeine and alcohol. For each dietary component, individual intake was standardized to a z-score by subtracting the global reference mean and dividing by the corresponding standard deviation derived from the CHINA-DII reference database established in the original development study. The z-scores were then converted to centered percentile scores to reduce skewness and minimize the effect of outliers. Each centered percentile value was multiplied by its corresponding inflammatory effect score derived from the CHINA-DII development model. The overall CHINA-DII score for each participant was obtained by summing the parameter-specific inflammatory scores across all included dietary components. The inflammatory effect score assigned to each of the 27 CHINA-DII components is provided in Supplementary Table S1 to enhance methodological transparency and facilitate replication of the CHINA-DII calculation.

### 2.6 Statistical analysis

Normality of continuous variables was assessed using descriptive statistics, including mean ± standard deviation (SD), skewness, and kurtosis. Continuous variables were presented as means ± SDs, while categorical variables were expressed as frequencies and percentages. Log transformation was performed in data with non-normally distribution. Comparisons between ischemic stroke cases and controls were performed using Student’s *t*-test for continuous variables and the chi-square test for categorical variables.

The CHINA-DII was analyzed both as a continuous variable and as tertiles based on its distribution in the study population. Multivariable logistic regression models were used to estimate odds ratios (ORs) and 95% confidence intervals (CIs) for the association between CHINA-DII and the risk of ischemic stroke. In Model 1, crude associations were assessed without adjustment. Model 2 was adjusted for potential confounders, including age, sex, education level, marital status, household income, occupation, BMI, physical activity, smoking status, alcohol consumption, hypertension, diabetes mellitus, and family history of stroke. Tests for linear trend across tertiles of CHINA-DII were conducted by assigning the median value of each tertile and modeling it as a continuous variable.

Subgroup analyses were further performed to evaluate potential effect modification by demographic and clinical characteristics, including age, sex, BMI, physical activity, smoking status, hypertension, diabetes mellitus, and family history of stroke. Interaction effects were assessed by including multiplicative interaction terms in the regression models.

A two-sided *p*-value < 0.05 was considered statistically significant. All statistical analyses were performed using SPSS software, version 25.0 (IBM Corp., Armonk, NY, USA).

## 3 Results

### 3.1 Characteristics of study subjects

A total of 584 participants were included in the present case–control study, comprising 292 patients with stroke and 292 controls. The baseline characteristics of the study population according to stroke status are presented in Table 1. Compared with controls, participants with stroke were significantly older (*p* < 0.001), had a higher prevalence of overweight/obesity (51.0% vs. 44.9%, *p* = 0.041), lower physical activity levels (*p* = 0.024), and lower educational attainment (*p* = 0.033). Stroke cases were also more likely to be current smokers (39.7% vs. 30.8%, *p* = 0.015), have hypertension (62.7% vs. 38.0%, *p* < 0.001), diabetes mellitus (25.0% vs. 16.8%, *p* = 0.006), and a family history of stroke (20.2% vs. 13.4%, *p* = 0.021) than controls.

**Table 1:** Characteristics of study participants by stroke status.

Variables	Total (*N* = 584)	Stroke (*N* = 292)	Controls (*N* = 292)	*p*-value
Age, years				
≤60	286 (49.0)	117 (40.1)	169 (57.9)	<0.001
>60	298 (51.0)	175 (59.9)	123 (42.1)	
Sex				
Male	258 (44.2)	125 (42.8)	133 (45.5)	0.59
Female	326 (55.8)	167 (57.2)	159 (54.5)	
BMI (kg/m^2^)				
Normal (<25)	304 (52.1)	143 (49.0)	161 (55.1)	0.041
Overweight/ obese (≥25)	280 (47.9)	149 (51.0)	131 (44.9)	
Physical activity (MET-h/week)				
Low (<20)	192 (32.9)	108 (37.0)	84 (28.8)	0.024
Moderate (20–40)	247 (42.3)	119 (40.8)	128 (43.8)	
High (>40)	145 (24.8)	65 (22.3)	80 (27.4)	
Marital status				
Married	522 (89.4)	267 (91.4)	255 (87.3)	0.087
Single/divorced/widowed	62 (10.6)	25 (8.6)	37 (12.7)	
Education level				
Primary school or below	196 (33.6)	111 (38.0)	85 (29.1)	0.033
Secondary school	168 (28.8)	84 (28.8)	84 (28.8)	
High school	112 (19.2)	50 (17.1)	62 (21.2)	
College/university	108 (18.5)	47 (16.1)	61 (20.9)	
Occupation				
Farmers/manual workers	180 (30.8)	98 (33.6)	82 (28.1)	0.112
Other occupations	214 (36.6)	100 (34.2)	114 (39.0)	
Homemaker/retired/unemployed	190 (32.5)	94 (32.2)	96 (32.9)	
Household income (RMB)				
<3,000	86 (14.7)	49 (16.8)	37 (12.7)	0.058
3,000–6,000	238 (40.8)	122 (41.8)	116 (39.7)	
>6,000	260 (44.5)	121 (41.4)	139 (47.6)	
Smoking				
Yes	206 (35.3)	116 (39.7)	90 (30.8)	0.015
No	378 (64.7)	176 (60.3)	202 (69.2)	
Alcohol drinking				
Yes	124 (21.2)	69 (23.6)	55 (18.8)	0.094
No	460 (78.8)	223 (76.4)	237 (81.2)	
Hypertension				
Yes	294 (50.3)	183 (62.7)	111 (38.0)	<0.001
No	290 (49.7)	109 (37.3)	181 (62.0)	
Diabetes mellitus				
Yes	122 (20.9)	73 (25.0)	49 (16.8)	0.006
No	462 (79.1)	219 (75.0)	243 (83.2)	
Family history of stroke				
Yes	98 (16.8)	59 (20.2)	39 (13.4)	0.021
No	486 (83.2)	233 (79.8)	253 (86.6)	
Overall CHINA-DII score	−1.52 ± 1.04	−1.16 ± 1.01	−1.88 ± 0.95	<0.001

Data were expressed as mean ± SD and percent (%) for continuous and categorical variables, respectively. *p*-values were calculated based on independent sample *t*-test or Chi-square test. DII, dietary inflammatory index; BMI, body mass index; MET, metabolic equivalent tasks.

### 3.2 Comparison of dietary nutrient intake between stroke cases and controls

Dietary nutrient intakes of study participants are shown in Table 2. Compared with controls, stroke patients had significantly lower intakes of dietary fiber (15.3 ± 6.4 vs. 17.5 ± 6.8 g/day, *p* < 0.001), vitamin C (95.8 ± 63.1 vs. 121.6 ± 72.4 mg/day, *p* < 0.001), *β*-carotene (3,891 ± 2,275 vs. 4,473 ± 2,461 μg/day, *p* = 0.004), and magnesium (276.8 ± 92.5 vs. 296.0 ± 98.7 mg/day, *p* = 0.018).

**Table 2:** Comparison of dietary nutrient intake between the stroke and control groups.

Nutrients	Total (*N* = 584)	Cases (*N* = 292)	Controls (*N* = 292)	*p*-value
Energy (kcal/day)	1832.6 ± 462.5	1848.2 ± 478.1	1817.0 ± 446.7	0.412
Protein (g/day)	71.8 ± 21.4	71.0 ± 22.3	72.6 ± 20.5	0.381
Carbohydrates (g/day)	248.7 ± 68.2	252.1 ± 70.4	245.3 ± 65.8	0.231
Fat (g/day)	63.5 ± 22.1	64.6 ± 23.0	62.4 ± 21.2	0.247
SFA (g/day)	18.2 ± 6.9	18.9 ± 7.1	17.5 ± 6.6	0.061
MUFA (g/day)	17.5 ± 6.7	17.7 ± 6.9	17.3 ± 6.5	0.514
PUFA (g/day)	11.8 ± 4.8	11.6 ± 4.9	12.0 ± 4.7	0.309
Cholesterol (mg/day)	328.4 ± 146.5	334.2 ± 149.8	322.6 ± 143.1	0.347
Dietary fiber (g/day)	16.4 ± 6.7	15.3 ± 6.4	17.5 ± 6.8	<0.001
Folate (μg/day)	284.6 ± 102.3	278.2 ± 100.6	291.0 ± 103.8	0.136
Vitamin A (μgRE/day)	653.6 ± 285.9	629.5 ± 279.8	677.9 ± 291.1	0.08
Vitamin B1 (mg/day)	1.03 ± 0.34	1.01 ± 0.35	1.05 ± 0.33	0.176
Vitamin B2 (mg/day)	1.24 ± 0.42	1.21 ± 0.43	1.27 ± 0.40	0.089
Vitamin B3 (mg/day)	18.9 ± 6.3	18.6 ± 6.5	19.2 ± 6.1	0.267
Vitamin B6 (mg/day)	1.31 ± 0.46	1.28 ± 0.45	1.34 ± 0.47	0.114
Vitamin B12 (μg/day)	4.18 ± 2.03	4.05 ± 2.11	4.31 ± 1.95	0.129
Vitamin C (mg/day)	108.7 ± 69.5	95.8 ± 63.1	121.6 ± 72.4	<0.001
Vitamin D (μg/day)	3.04 ± 1.62	2.95 ± 1.58	3.13 ± 1.65	0.181
Vitamin E (mg/day)	12.7 ± 5.1	12.4 ± 5.2	13.0 ± 4.9	0.162
β-carotene (μg/day)	4,182 ± 2,384	3,891 ± 2,275	4,473 ± 2,461	0.004
Iron (mg/day)	19.2 ± 6.5	18.9 ± 6.7	19.5 ± 6.2	0.268
Zinc (mg/day)	12.8 ± 4.1	12.6 ± 4.2	13.0 ± 4.0	0.243
Magnesium (mg/day)	286.4 ± 96.2	276.8 ± 92.5	296.0 ± 98.7	0.018
Selenium (μg/day)	58.7 ± 23.8	58.0 ± 24.2	59.4 ± 23.4	0.485
Calcium (mg/day)	612.5 ± 224.1	595.2 ± 218.3	629.8 ± 228.7	0.067
Caffeine (mg/day)	24.7 ± 18.9	23.5 ± 18.2	25.9 ± 19.5	0.128
Alcohol (g/day)	8.9 ± 12.7	9.8 ± 13.5	8.0 ± 11.8	0.091

The variables are presented as mean ± SD and compared using the *t*-test.

### 3.3 Association between CHINA-DII and risk of stroke

The associations between CHINA-DII and stroke risk are summarized in Table 3. In the crude model, each one-unit increase in CHINA-DII score was associated with a 34% higher odds of stroke (OR: 1.34; 95% CI: 1.18–1.52; *p* < 0.001). After adjustment for potential confounders, this association remained significant (OR: 1.27; 95% CI: 1.10–1.46; *p* = 0.001).

**Table 3:** Logistic regression analysis for the association between CHINA-DII score and risk of stroke.

Variables	Cases/controls	Model 1* OR (95% CI)	*p*-value	Model 2** OR (95% CI)	*p*-value
CHINA-DII (continuous)	—	1.34 (1.18–1.52)	<0.001	1.27 (1.10–1.46)	0.001
CHINA-DII tertiles					
T1	71/124	1.00	—	1.00	—
T2	96/98	1.71 (1.11–2.60)	0.019	1.54 (0.98–2.38)	0.062
T3	125/70	3.12 (2.03–4.79)	<0.001	2.41 (1.51–3.84)	<0.001
P for trend	—	—	<0.001	—	0.001

*Model 1: crude model. **Model 2: adjusted for age, sex, education level, marital status, household income, occupation, hypertension, and diabetes, smoking status, alcohol consumption, body mass index, physical activity and family history of stroke.

DII, dietary inflammatory index; OR, odds ratio; CI, confidence interval.

When CHINA-DII was analyzed in tertiles, participants in the highest tertile (T3) had significantly greater odds of stroke compared with those in the lowest tertile (T1) in both crude (OR: 3.12; 95% CI: 2.03–4.79; *p* < 0.001) and adjusted models (OR: 2.41; 95% CI: 1.51–3.84; *p* < 0.001).

### 3.4 Subgroup analyses

Subgroup analyses stratified by demographic and clinical characteristics are presented in Table 4. The positive association between CHINA-DII and stroke risk was generally consistent across most subgroups. The association appeared stronger among older participants (>60 years), individuals with overweight/obesity, smokers, and those with hypertension. A significant interaction was observed for physical activity (P for interaction = 0.041), indicating that the association between CHINA-DII and stroke risk was more pronounced among participants with low or moderate physical activity (OR: 2.71; 95% CI: 1.63–4.50; *p* < 0.001) than among those with high physical activity (OR: 1.68; 95% CI: 0.81–3.47; *p* = 0.164).

**Table 4:** Subgroup analysis of CHINA-DII and stroke risk by demographic characteristics.

Subgroups	OR (95% CI)	*p*-value	P for Interaction
Age, years			
≤60	2.12 (1.18–3.82)	0.012	0.184
>60	2.63 (1.47–4.70)	0.001	
Sex			
Male	2.35 (1.39–3.98)	0.002	0.427
Female	2.49 (1.32–4.68)	0.005	
BMI (kg/m^2^)			
<24	2.08 (1.17–3.70)	0.013	0.093
≥24	2.84 (1.55–5.19)	0.001	
Physical activity			
Low/moderate	2.71 (1.63–4.50)	<0.001	0.041
High	1.68 (0.81–3.47)	0.164	
Smoking			
No	2.19 (1.29–3.71)	0.004	0.228
Yes	2.74 (1.39–5.39)	0.004	
Family history of stroke			
Yes	1.65 (0.96–2.18)	0.098	0.31
No	1.51 (0.96–2.44)	0.064	
Hypertension			
No	1.89 (1.01–3.52)	0.046	0.067
Yes	2.88 (1.61–5.16)	<0.001	
Diabetes mellitus			
No	2.31 (1.42–3.76)	0.001	0.301
Yes	1.76 (0.93–3.10)	0.088	

Each stratified model was adjusted for age, sex, BMI, physical activity, smoking status, family history of stroke, hypertension and diabetes status, except for the stratification variable itself. DII, dietary inflammatory index; BMI, body mass index; OR, odds ratio; CI, confidence interval.

## 4 Discussion

This study aimed to investigate the association between the newly developed CHINA-DII and the risk of ischemic stroke. The primary objective was to determine whether a more pro-inflammatory diet, reflected by higher CHINA-DII scores, is associated with an increased likelihood of ischemic stroke. Our findings demonstrated that higher CHINA-DII scores were significantly associated with increased odds of ischemic stroke, even after adjusting for a wide range of demographic, socioeconomic, and clinical confounders. These findings highlight the potential role of diet-related inflammation in the pathogenesis of ischemic stroke and are consistent with the hypothesis that anti-inflammatory dietary patterns may be associated with a lower risk of stroke. However, given the observational case–control design, no causal inference can be drawn.

To our knowledge, no previous study has directly examined the association between CHINA-DII and ischemic stroke. However, our findings are consistent with prior research using the original DII, which has shown that pro-inflammatory diets are associated with an increased risk of cardiovascular diseases, including stroke (12, 13). Several large observational studies have reported that higher DII scores are linked to elevated levels of inflammatory biomarkers and a greater risk of stroke and other cardiometabolic conditions (18, 19). These findings support the hypothesis that chronic low-grade inflammation, influenced by dietary factors, plays a central role in vascular dysfunction and atherosclerosis, contributing to stroke development.

Emerging evidence links higher CHINA-DII scores to adverse health outcomes. In a Fujian, China case–control study of gastric cancer (GC), higher CHINA-DII was associated with significantly increased GC risk, and each SD increase in CHINA-DII raised GC odds by approximately 26% (10). Similarly, in a Chinese cohort of cardiovascular patients, the highest dietary inflammation group (China-DII ≥ 3.0) had dramatically higher odds of stroke and cardiovascular mortality compared to the lowest-risk group (11). However, CHINA-DII research is still nascent, focused mainly on China-specific outcomes (gastric cancer, CVD). No prospective CHINA-DII studies or large meta-analyses have yet been published. Future research should apply CHINA-DII to other diseases (diabetes, metabolic syndrome, and other cancers) and in longitudinal cohorts. Although the CHINA-DII was recently developed and demonstrated promising performance in the Chinese population, additional external validation studies are warranted to evaluate its applicability, reproducibility, and predictive ability across different geographic regions, ethnic groups, and dietary patterns. Such studies will help determine the generalizability and broader utility of the CHINA-DII in diverse populations.

Beyond epidemiological associations, biological evidence underlines how dietary inflammatory agents might increase the risk of ischemic stroke. Dietary patterns high in pro-inflammatory components (saturated fat, added sugars, low fiber) chronically activate innate immunity and endothelial stress. In particular, excess intake of saturated fatty acids and refined carbohydrates promotes gut microbiota dysbiosis and enhances lipopolysaccharide (LPS)-mediated activation of Toll-like receptor 4 (TLR4), which in turn amplifies the canonical nuclear factor kappa B (NF-κB) signaling pathway. This activation leads to increased production of pro-inflammatory cytokines, including interleukin-6 (IL-6) and tumor necrosis factor-alpha (TNF-*α*), and primes the NOD-like receptor family pyrin domain-containing 3 (NLRP3) inflammasome through NF-κB–mediated transcriptional upregulation of the NLRP3 gene (20). This induces ROS generation, leading to lipid peroxidation, damaging membranes, and inactivating glutathione/GPX4. The result is ferroptotic neuronal death and endothelial injury in ischemic brain. In parallel, chronic inflammation upregulates endothelial adhesion molecules, tissue factor and coagulation, promoting thrombosis (21). Anti-inflammatory nutrients (omega-3 s, polyphenols, vitamins C/E, Se, Zn) oppose these mechanisms by blocking NF-κB/NLRP3 signaling and bolstering antioxidant defenses (e.g., GPX4), highlighting potential intervention points (22, 23). It should be noted that these biological mechanisms are proposed based on evidence from previous experimental and clinical studies and were not directly evaluated in the present study. Therefore, they should be interpreted as biologically plausible explanations for the observed association rather than direct mechanistic findings.

This study has several notable strengths. To our knowledge, it is the first to investigate the association between the newly developed CHINA-DII and the risk of ischemic stroke, providing novel evidence on diet-related inflammation in a Chinese population. Dietary intake was assessed using a validated questionnaire, allowing for a comprehensive evaluation of habitual dietary patterns. Furthermore, we adjusted for a wide range of potential confounders, including demographic, socioeconomic, and clinical factors, and employed a matched case–control design, which enhances internal validity and reduces selection bias. Collectively, these methodological strengths strengthen the robustness and credibility of our findings.

However, several limitations should be considered. First, dietary intake was self-reported using a food frequency questionnaire and may therefore be subject to recall bias and measurement error. Although trained interviewers administered the questionnaire, participants with ischemic stroke may have recalled their pre-stroke dietary habits differently from controls, potentially resulting in differential misclassification of dietary exposure. Second, owing to the observational case–control design, causal relationships cannot be established, and residual confounding from unmeasured or imperfectly measured variables may still exist despite adjustment for a wide range of potential confounders. Third, reverse causation cannot be excluded, as dietary habits may have changed before stroke diagnosis due to preclinical symptoms or underlying health conditions. Therefore, prospective cohort studies with repeated dietary assessments are warranted to confirm the observed associations.

## 5 Conclusion

In this multi-centered case–control study, we demonstrated that higher CHINA-DII scores, reflecting a more pro-inflammatory diet, are significantly associated with an increased risk of ischemic stroke in a Chinese population. These findings support the role of diet-induced inflammation in stroke pathogenesis and highlight the importance of dietary patterns as a modifiable risk factor. Promoting anti-inflammatory dietary habits may represent a practical strategy for stroke prevention. Further prospective studies and mechanistic research are warranted to confirm these findings and clarify the causal relationship between dietary inflammation and stroke risk.

## Data Availability

The raw data supporting the conclusions of this article will be made available by the authors, without undue reservation.
