# Supplementary material for: Association between the newly proposed Chinese dietary inflammatory index and risk of ischemic stroke: a multi-centered case—control study
Source: Front Nutr. 2026 Jul 22;13:1861328. doi: 10.3389/fnut.2026.1861328 (PMC13437406; doi:10.3389/fnut.2026.1861328)
Supplement: Supplementary file 1 [file Table_1.docx]

**Association between the newly proposed Chinese Dietary Inflammatory Index and risk of ischemic stroke: a multi-centered case - control study**

| **Supplementary Table S1. Dietary components included in the China Dietary Inflammatory Index (CHINA-DII) and their inflammatory effect scores.** | |
| --- | --- |
| **Dietary component** | **Inflammatory effect score*** |
| Energy | +0.180 |
| Protein | −0.021 |
| Carbohydrate | +0.097 |
| Total fat | +0.298 |
| Cholesterol | +0.110 |
| Saturated fatty acids | +0.373 |
| Monounsaturated fatty acids | −0.009 |
| Polyunsaturated fatty acids | −0.337 |
| Dietary fiber | −0.663 |
| Vitamin A | −0.401 |
| β-Carotene | −0.584 |
| Vitamin B1 (Thiamine) | −0.098 |
| Vitamin B2 (Riboflavin) | −0.068 |
| Vitamin B6 | −0.365 |
| Vitamin B12 | −0.106 |
| Vitamin C | −0.424 |
| Vitamin E | −0.419 |
| Niacin | −0.246 |
| Folate | −0.190 |
| Iron | +0.032 |
| Magnesium | −0.484 |
| Zinc | −0.313 |
| Selenium | −0.191 |
| Alcohol | −0.278 |
| Tea | −0.536 |
| Garlic | −0.412 |
| Ginger | −0.453 |
| CHINA-DII, China Dietary Inflammatory Index; DII, Dietary Inflammatory Index. ***** Inflammatory effect scores were obtained from the published CHINA-DII methodology proposed by Zhang et al*.* | |
